# Editorial: Trace elements in the environment: Biogeochemical cycles and bioremediation

**DOI:** 10.3389/fmicb.2022.1056528

**Published:** 2022-12-13

**Authors:** Jian Chen, Ximei Xue, Mingshun Li

**Affiliations:** ^1^Department of Cellular Biology and Pharmacology, Herbert Wertheim College of Medicine, Florida International University, Miami, FL, United States; ^2^Key Laboratory of Urban Environment and Health, Institute of Urban Environment, Chinese Academy of Sciences, Xiamen, China; ^3^State Key Laboratory of Agricultural Microbiology, College of Life Science and Technology, Huazhong Agricultural University, Wuhan, China

**Keywords:** trace element, bioavailability, biotransformation, bioremediation, biosensor

Trace elements are chemical elements present in living tissues in very small amounts and many of them have no biological function and not essential to humans. But it becomes toxic to life when uptake concentrations exceed acceptable risk-based levels (Egorova and Ananikov, 2017). Natural and anthropogenic activities introduce trace elements into the environment and result in soil, water and food contaminations. Microbe-mediated processes, such as methylation, and demethylation contribute to the trace elements biogeochemical cycles. In turn, trace elements also alter microbial community structure, which potentially changes their behavior in processes such as biotransformation and bio-precipitation (Figure 1). Therefore, knowledge of the crucial roles of microbes and functional genes involved in trace elements bio-cycle is important for its exploitation for bioremediation and bio-sensing. Bioremediation has received wide attention due to its advantages in providing an effective and affordable technology and eco-friendly *in situ* sustainable approach for toxic trace elements removal (Valls and de Lorenzo, 2002). Mealtime, development of better methods for detection of them in environment is essential. Microorganisms and all cells keep trace element homeostasis by highly specialized metal sensing transcriptional regulators. A specific biosensor is a combination of a biological sensing element and a transducer (Zeng et al., 2021). When trace element is present, the regulatory protein interacts with element and activates the reporter gene expression (Figure 1). This Research Topic is to explore the microbe-mediated trace elements biogeochemical cycle, bioremediation and bio-sensing, including the development of analytical techniques employed to study trace element transformations.

**Figure 1:**
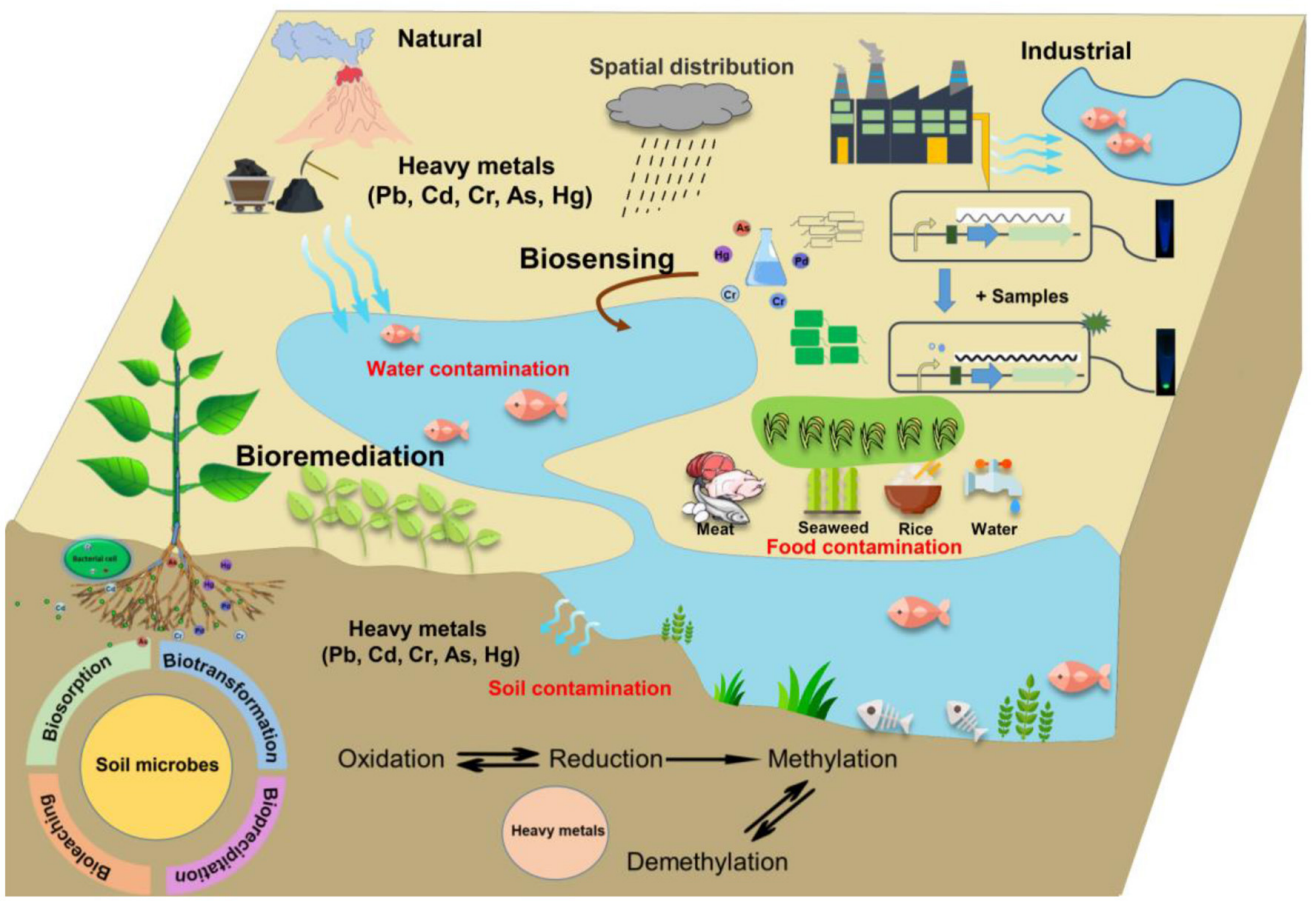
The global trace elements cycle in the environment and its bioremediation and biosensing.

Sustainable arsenic (As)-remediation technology to make production of arsenic-safe water is currently a challenge. In this Research Topic, Yuan et al. developed a bioelectrochemical system (BES) and investigated its arsenic removal capability from groundwater. When BES was operated with 30 mA currents (I_cell_), 90 ± 3% of total. As was removed from water due to microbial electrosorption in the sludge. Two bacteria of As(V)-reducing bacteria and sulfate-reducing bacteria coexist in the sludge of cathode chamber. This BES-based technology exhibits a high efficiency for As(V) removal, without any organic or chemical additive required. It is an eco-friendly technique that can be potentially exploited for As-contaminated groundwater remediation. Chromium (Cr)(VI) is also a well-known environmental contaminant (Sharma et al., 2022). Reduction of Cr(VI) to Cr(III) with less toxicity and mobility is an important process for Cr remediation. Rahman and Thomas comprehensively reviewed microbial bio-reduction of Cr(VI) and the contributions of various chemicals, such as electron donors, mediators, and other chemical additives in Cr(VI) removal. Organic molecules, such as glucose, are the most common electron donors for Cr(VI) reduction. But non-fermentable substrates, such as citrate, exhibit lower Cr(VI) removal rates. Electron transfer competence for Cr(VI) reduction also differs in various mediator compounds. Chemical additives implementation also affects reduction efficiency. The availability of various chemicals limits microbial Cr(VI) reduction in large-scale. Developing eco-friendly technologies that use unexploited potential of the natural ecosystems for Cr(VI) remediation is a necessity. Compared to bacteria bioremediation, a unique plant-microbe combination offers the potential for achieving greater remediation efficiency (Ojuederie and Babalola, 2017). Niu et al. showed *S. integra*-indigenous bacteria combination has great application in the remediation of Pb-contaminated soil by adjusting soil properties. *S. integra* absorbed the highest Pb in root under high Pb treatment. *S. integra* cultivation decreased the total Pb but increased soluble Pb in bulk soil and rhizosphere under the low Pb treatment. High-throughput sequencing results showed that indigenous bacteria, such as Proteobacteria and Actinobacteria were promoted by root activity and/or Pb stress. Results indicate microbial community composition can be shifted by adjusting soil properties and indigenous bacteria can be utilized in plant-microbe combined remediation.

In aquatic systems, mercury contamination poses a serious environmental problem (Amos et al., 2013). Algae *Euglena Gracilis* is an ideal candidate to explore the mechanisms involved in Hg resistance. Mangal et al. used a combination of Fourier Transform Ion Cyclotron ResonanceMass Spectrometry and RNA-sequencing to identify the transcriptomic and metabolomic changes in intracellular or extracellular of *E. Gracilis* after mercury exposure. The transcripts and metabolites involved in cellular responses to chemical stress and reactive oxygen species were significantly upregulated. It is novel to identify changes in the metabolome of *E. gracilis* by combination of RNA-sequencing, gene expression and transcriptomic analyses, which helps to explore the specific cellular responses and mechanisms for Hg tolerance. To evaluate Hg bioavailability, Hui et al. developed a biosensor with dual-sensing constructs by utilizing two sensory regulatory proteins with cadmium-responsive regulator (CadR) and mercury-responsive regulator (MerR) separately, and two reporters with green fluorescent protein and red fluorescent protein separately. Bioavailable Hg(II) (0–5 μM) and Cd(II) (0–200 μM) could be quantitatively determined using double-color fluorescence. This robust biosensor can distinguish coexisting Hg(II) and Cd(II), which will be valuable, practical, and helpful in simultaneously monitoring multiple heavy metal pollutants in real world applications. Using the same strategies, Hui et al. also developed another dual-sensing biosensor specifically for Cd(II) detection. In this biosensor, the genetic circuit is composed of CadC and CadR as separate metal sensory elements. It successfully distinguished Cd(II) from non-target-responsive elements Pb(II) or Hg(II) by producing two bio-sensing signals. It maintained high sensitivity of Cd(II), but more specifically for Cd(II), thereby serving as a quantitative and effective biosensor for bioavailable Cd(II) detection. These findings illustrate that combination of multiple sensory elements is a new strategy to improve the performance of biosensor devices.

The research shown in this topic develops advanced approaches for trace elements bioremediation, such as exploiting indigenous bacteria and increasing elements bioavailability by reduction enhancement, and bio-sensing, such as using dual-sensing bio-reporters. It provides efficient strategies to solve the environmental problems of trace elements contamination. Meantime, great challenges stand in the way of sensing and removing toxic trace elements in the environment. This complexity of environmental samples hinders field analysis by introducing bias into the analysis process, such as matrix interferences, which make the results unreliable (Galuszka et al., 2015). There is still a gap in moving our laboratories to the field. And bioremediation research must be capable of being scaled up, microbial processes in the polluted environment, and bioavailability of trace elements need to be well investigated. Therefore, developing advanced techniques for trace elements detection, identification and removal remain principal goals in future research.

## Author contributions

All authors listed have made a substantial, direct, and intellectual contribution to the work and approved it for publication.
